# Prostate-Specific Membrane Antigen Uptake and Survival in Metastatic Castration-Resistant Prostate Cancer

**DOI:** 10.3389/fonc.2021.630589

**Published:** 2021-02-18

**Authors:** Panagiotis J. Vlachostergios, Muhammad Junaid Niaz, Michael Sun, Seyed Ali Mosallaie, Charlene Thomas, Paul J. Christos, Joseph R. Osborne, Ana M. Molina, David M. Nanus, Neil H. Bander, Scott T. Tagawa

**Affiliations:** ^1^ Division of Hematology and Medical Oncology, Department of Medicine, Weill Cornell Medicine, New York, NY, United States; ^2^ Department of Urology, Weill Cornell Medicine, New York, NY, United States; ^3^ Division of Molecular Imaging and Therapeutics, Department of Radiology, Weill Cornell Medicine, New York, NY, United States; ^4^ Division of Biostatistics and Epidemiology, Department of Healthcare Policy & Research, Weill Cornell Medicine, New York, NY, United States; ^5^ Sandra and Edward Meyer Cancer Center, Weill Cornell Medicine, New York, NY, United States

**Keywords:** prostate specific membrane antigen, metastatic castration resistant prostate cancer, overall survival, nuclear imaging, positron emission tomography, single photon emission computed tomography

## Abstract

**Background:**

Prostate-specific membrane antigen (PSMA) imaging has been suggested as highly sensitive modality for detection of metastases in patients with biochemically recurrent or advanced prostate cancer (PCa). PSMA expression is associated with grade and stage and has a relationship with androgen receptor signaling. The aim of this study was to evaluate the prognostic utility of radiographic PSMA expression in men with metastatic castration-resistant prostate cancer (mCRPC).

**Methods:**

Patients with mCRPC and available baseline PSMA imaging were studied. Images by planar/single-photon emission computed tomography (SPECT) or positron emission tomography (PET)/CT were reviewed. Planar/SPECT images were scored semi-quantitatively and PET/CT scored quantitatively with comparison of tumor uptake to liver uptake on a scale of 0–4 in order to determine an imaging score (IS). The IS (high: 2–4 versus low: 0–1), subsequent receipt of life-prolonging systemic therapies (taxane chemotherapy, potent androgen receptor pathway inhibitors, sipuleucel-T, and radium-223), and the CALGB prognostic risk stratification of patients were analyzed according to overall survival (OS) in univariate and multivariate Cox’s proportional hazards models.

**Results:**

High PSMA expression (IS 2–4) was found in 179 (75.21%) patients, and 59 (24.79%) patients had low PSMA uptake. The median OS of the entire cohort was 16.8 (95%CI: 14.9–19.3) months. Patients with a high IS had a significantly shorter OS of 15.8 (95%CI 13.0–18.1) months compared to those with low expression [22.7 (95%CI: 17.7–30.7) months, p = 0.002]. After accounting for use of life-prolonging therapies (p<0.001) and CALGB prognostic groups (p = 0.001), high PSMA IS emerged as an independent prognostic factor for OS [HR(95%CI): 1.7 (1.2–2.2); p = 0.003].

**Conclusion:**

Presence of high radiographic PSMA expression on SPECT or PET/CT may portend a poor prognosis in patients with mCRPC treated with standard systemic therapies. This provides implications for therapeutic targeting of PSMA-avid disease as a means to improve outcomes.

## Introduction

Prostate cancer (PCa) is the leading non-cutaneous malignancy among adult males in United States. It accounts for about 20% of the newly diagnosed cancers amongst U.S. men each year. In 2020, almost 191,930 new cases of prostate cancer will be diagnosed and about 33,330 men are expected to die (1).

Prostate specific membrane antigen (PSMA) is a 100-kD type 2 integral transmembrane metalloenzyme/glycoprotein that has emerged as a key target in the diagnosis and treatment of metastatic castration-resistant PCa (2, 3). While restricted to apical epithelium of secretory ducts within normal prostate glands, PSMA is significantly upregulated in PCa (2, 3). Expression levels increase with tumor grade/de-differentiation, development of castration resistance, and dysregulation of androgen receptor signaling (2, 3). PSMA expression is downregulated by androgens, and conversely, anti-androgen therapy increases its expression (4, 5). Interestingly, higher PSMA expression in prostate tumor tissues has been associated with lethal PCa and may predict disease recurrence following curative therapy for PCa (6, 7).

The development of imaging ligands directed towards PSMA, the most commonly used being the urea-based PSMA-11 (2), conjugated to radiotracers (frequently ^68^Ga), has offered a noninvasive way of target assessment for diagnostic or/and therapeutic purposes. Compared to traditional imaging modalities, including bone and CT scans, PSMA-PET scans have shown greater efficacy in detecting early recurrent disease, bone metastases, and small lymph node metastases (2). In a randomized study of high-risk PCa patients, PSMA PET-CT demonstrated superior accuracy in detecting metastatic disease compared to conventional methods as first-line imaging (8). The feasibility and value of single photon emission computed tomography (SPECT) for assessment of metastatic lesions in mCRPC patients undergoing ^177^Lu-PSMA radioligand therapy has also been demonstrated (9). Besides their use for staging purposes, conventional imaging modalities using bone, CT, and MRI scans have not been shown to predict patient outcomes.

In this study, we examined the prognostic role of baseline PSMA-SPECT and PSMA-PET in patients with mCRPC who were treated with various life-prolonging therapies and underwent baseline PSMA imaging.

## Materials and Methods

### Patient Selection

Patients with progressive metastatic castration-resistant prostate cancer (mCRPC) enrolled in PSMA imaging studies were analyzed (10–18). Participants underwent planar gamma camera imaging (^111^In-J591 and/or ^177^Lu-J591) and/or ^68^Ga-PSMA-11 PET/CT.

### Image Acquisition

Planar anterior and posterior gamma camera images were obtained on a GE SPECT/CT system 5–7 days after administration of ^177^Lu-J591 or ^111^In-J591. SPECT combined with low-dose CT of an area of interest was also performed but not used for the visual score assessment. For PSMA-PET imaging, the small molecular compound PSMA-11 (ABX Advanced Biochemical Compounds, Radeberg, Germany), also known as PSMA-HBED-CC, was utilized. PSMA-11 was conjugated to ^68^Ga. Study subjects received 5 mCi of ^68^Ga-PSMA-11 during the screening visit and prior to administration of radionuclide therapy. PET-CT, from vertex of skull to mid-thighs, was obtained 1–3 h after completion of infusion.

### Image Analysis

Anterior and posterior planar images and PSMA-PET scans were reviewed independently by two nuclear medicine radiologists. For planar imaging, the three lesions with the highest uptake were scored on a 5-point visual scale from 0 to 4 as follows: 0 (tumor undetectable), 1 (faint tumor activity detectable), 2 (strong tumor activity but less than liver activity), 3 (tumor activity equal to liver), and 4 (tumor greater than liver activity) (12–15) (**Table 1**). PET images were scored by averaging SUVmax of the five lesions with highest uptake and then comparing that value with liver SUVmean. 1=SUVmax < liver mean SUV, 2=SUVmax 1-2.5x liver SUV, 3=SUVmax 2.5-5x liver SUV, 4=SUVmax > 5x liver SUV (18) (**Table 2**).

**Table 1:** Imaging score (IS) assessment on SPECT.

IS	SPECT imaging (Top 3 lesions with highest uptake)
**0**	Undetectable activity
**1**	Faint activity
**2**	Lesions’ activity < liver
**3**	Lesions’ activity = liver
**4**	Lesions’ activity > liver

**Table 2:** Imaging score (IS) assessment on PET.

IS	PET imaging (Top 5 lesions with highest SUVmax)
**0**	Average SUV_max_ < blood pool
**1**	Average SUV_max_ < liver SUV_mean_
**2**	Average SUV_max_ 1-2.5x liver SUV_mean_
**3**	Average SUV_max_ 2.5-5x liver SUV_mean_
**4**	Average SUV_max_ > 5x liver SUV_mean_

### Statistical Analysis

Baseline clinical characteristics, including CALGB (Halabi) prognostic criteria (lymph node/osseous/visceral metastases, opioid analgesic use, ECOG performance status, serum lactate dehydrogenase, hemoglobin, albumin, alkaline phosphatase, PSA) (19), were documented at time of imaging. The Kaplan-Meier method was used to compare imaging scores (IS) with overall survival (OS), measured as the time from PSMA imaging until death. Multivariable Cox proportional hazards regression analysis was used to control for Halabi prognostic variables and receipt of subsequent life-prolonging systemic therapies in assessing OS (taxanes, potent androgen receptor pathway inhibitors, including abiraterone and enzalutamide, sipuleucel-T, radium-223). The cut-off IS of 2 (high: 2–4 versus low: 0–1), used for Kaplan Meier and multivariate survival analyses, was pre-specified, as previously described (12–15). Statistical significance was set at the 0.05 alpha level. Analyses were performed using STATA version 15.0 (StataCorp).

## Results

Two hundred thirty-eight men with metastatic CRPC, median PSA 73.55 ng/dl (0.49–2746) and median age 70.5 (44–93), were studied. One hundred eighty-seven patients underwent planar/SPECT scan and 51 had PET/CT (**Table 3**). Two-thirds of patients (61%) had received prior taxane and 40% potent androgen receptor pathway inhibitors. One-third (33%) was treated after PSMA imaging with taxanes and 16% with potent androgen receptor pathway inhibitors. A minority received sipuleucel-T (pre: 12%, post: 2%) or radium-223 (pre:7%, post: 2.5%) before or following PSMA scan (**Table 3**). Fifty-nine (24.8%) patients had low PSMA expression by imaging (IS 0-1), whereas 179 (75.2%) had high PSMA expression (IS 2-4).

**Table 3:** Clinical characteristics of patients with mCRPC who underwent PSMA imaging.

Baseline Characteristics (N = 238)	
**Age (years)**	
**Median (Range)**	70.5 (44–93)
**Gleason sum, *n* (%)**	
**5**	4 (1.7%)
**6**	32 (13.4%)
**7**	62 (26.1%)
**8**	49 (20.6%)
**9**	73 (30.7%)
**10**	6 (2.5%)
**Not reported**	12 (5%)
**Sites of Metastases, *n* (%)**	
**Bone**	206 (86.6%)
**Lymph Node**	127 (53.4%)
**Liver**	19 (7.9%)
**Lung**	39 (16.4%)
**ECOG Status, *n* (%)**	
**0**	21 (8.8%)
**1**	190 (79.8%)
**2**	22 (9.2%)
**Not reported**	5 (2.1%)
**CALGB (Halabi) Prognostic Category, *n* (%)**	
**High**	147 (61.8%)
**Intermediate**	76 (31.93%)
**Low**	9 (3.78%)
**Not reported**	6 (2.52%)
**Narcotics Use, *n* (%)**	
**Yes**	75 (31.51%)
**No**	159 (66.81)
**Not reported**	4 (1.68%)
**Pre Scan Therapy, *n* (%)**	
**Taxanes**	145 (60.9%)
**ARPI**	96 (40.3%)
**Sip-T**	28 (11.8%)
**Ra 223**	17 (7.1%)
**Post Scan Therapy, *n* (%)**	
**Taxanes**	78 (32.8%)
**ARPI**	39 (16.4%)
**Sip-T**	4 (1.7%)
**Ra 223**	6 (2.5%)
**PSA (ng/mL)**	
**Median (Range)**	49.5 (1.93–3533.5)
**Hemoglobin (g/dL)**	
**Median (Range)**	12.4 (7.7–16.3)
**Albumin (g/dL)**	
**Median (Range)**	3.7 (2.2–4.9)
**Alkaline Phosphatase (U/L)**	
**Median (Range)**	99 (22–1321)
**Lactate Dehydrogenase (U/L)**	
**Median (Range)**	215 (99–1083)

**Figure 1:**
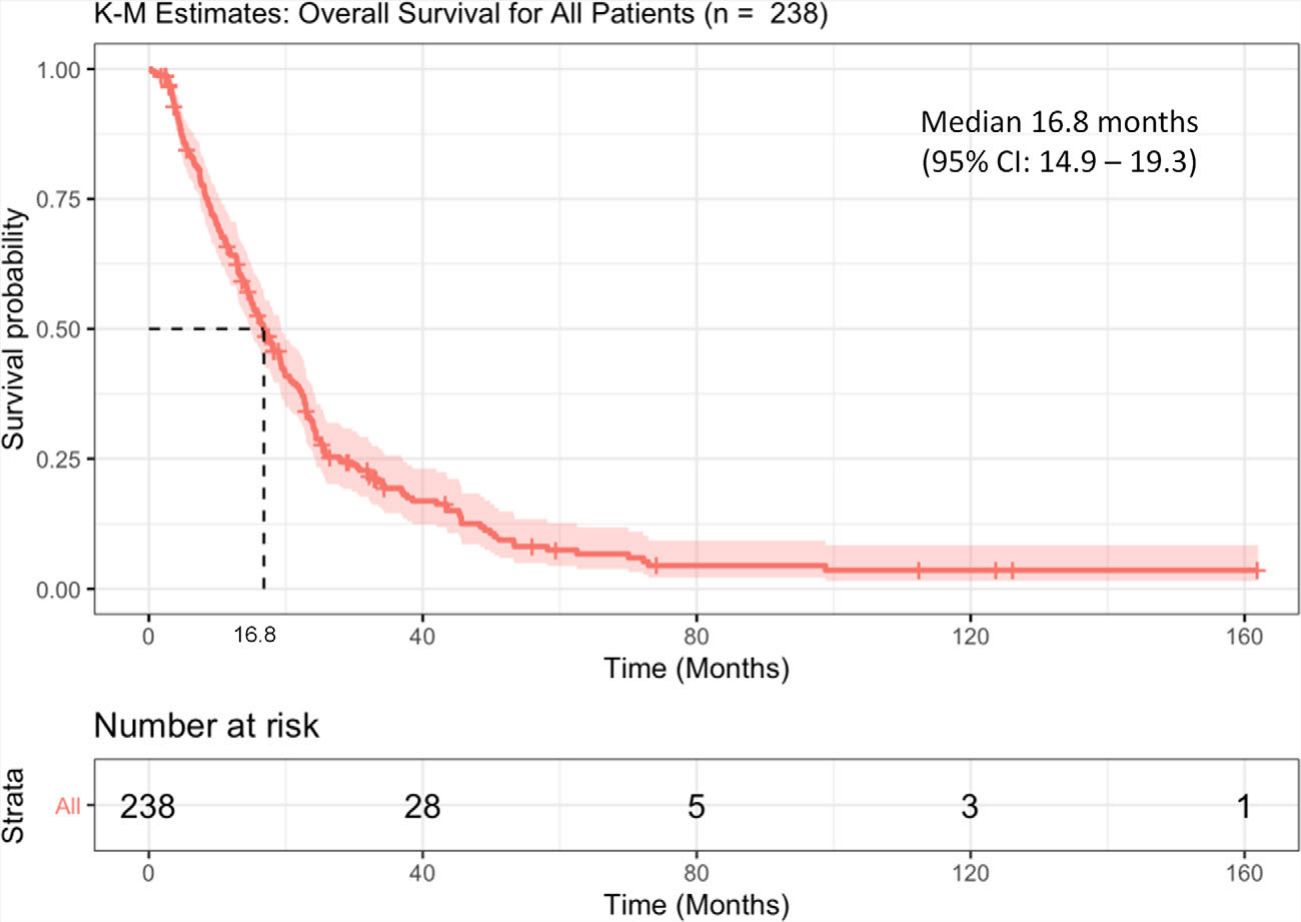
Kaplan Meier curve of overall survival (OS) of the entire cohort.

**Figure 2:**
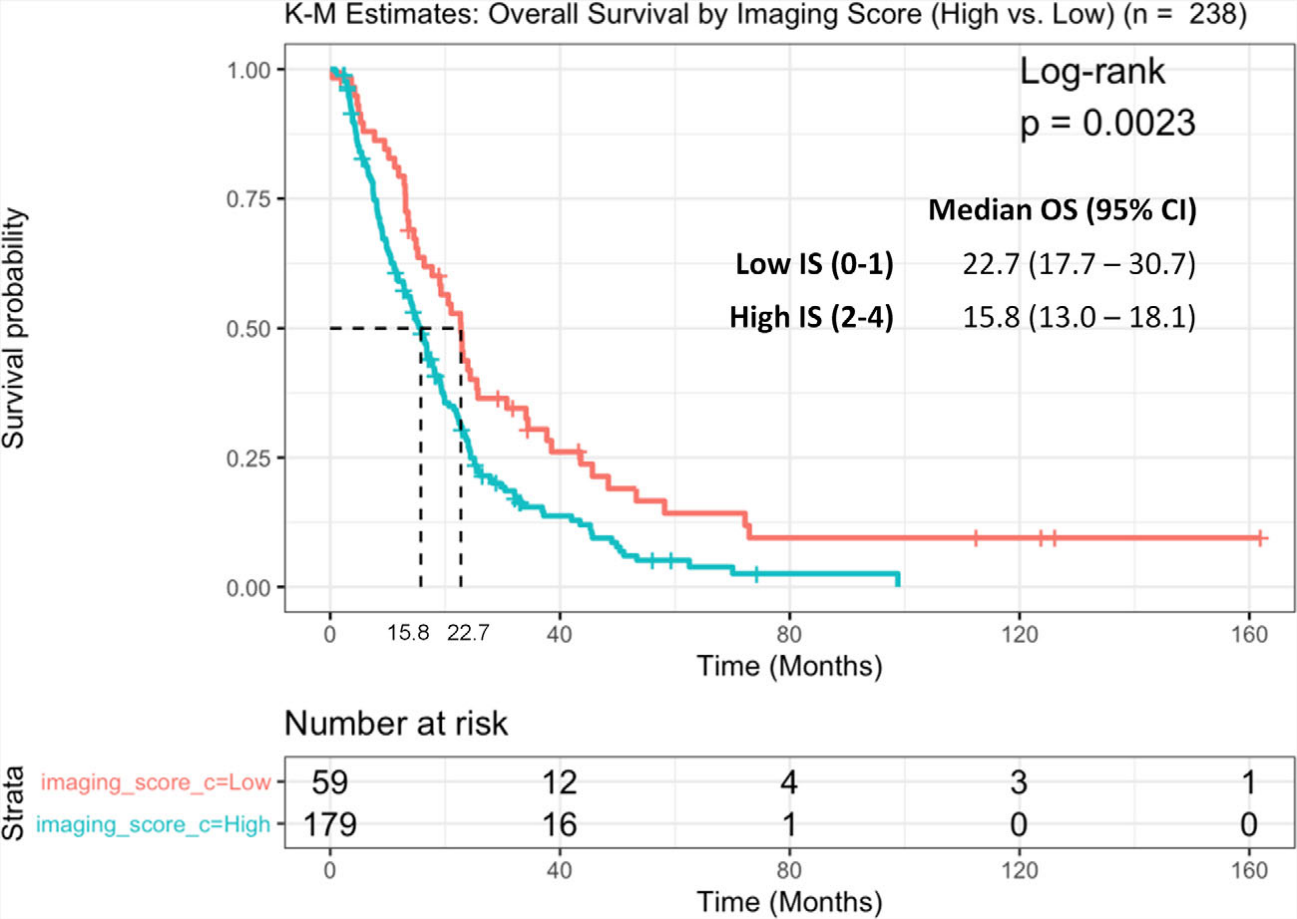
Kaplan Meier curve of overall survival (OS) between patients with high vs. low imaging score (IS).

The median survival (OS) of the entire cohort (from the time of PSMA imaging) was 16.8 months (95%CI: 14.9–19.3) (**Figure 1**); 147 (61.8%) patients had high-risk Halabi score, 76 (31.9%) were intermediate-risk, and 9 (3.8%) low-risk. Patients with low IS had a median OS of 22.7 months (95%CI: 17.7–30.7), whereas patients with high IS had a significantly shorter OS of 15.8 months (95%CI: 13.0–18.1, log-rank p = 0.002 (**Figure 2**).

**Figure 3:**
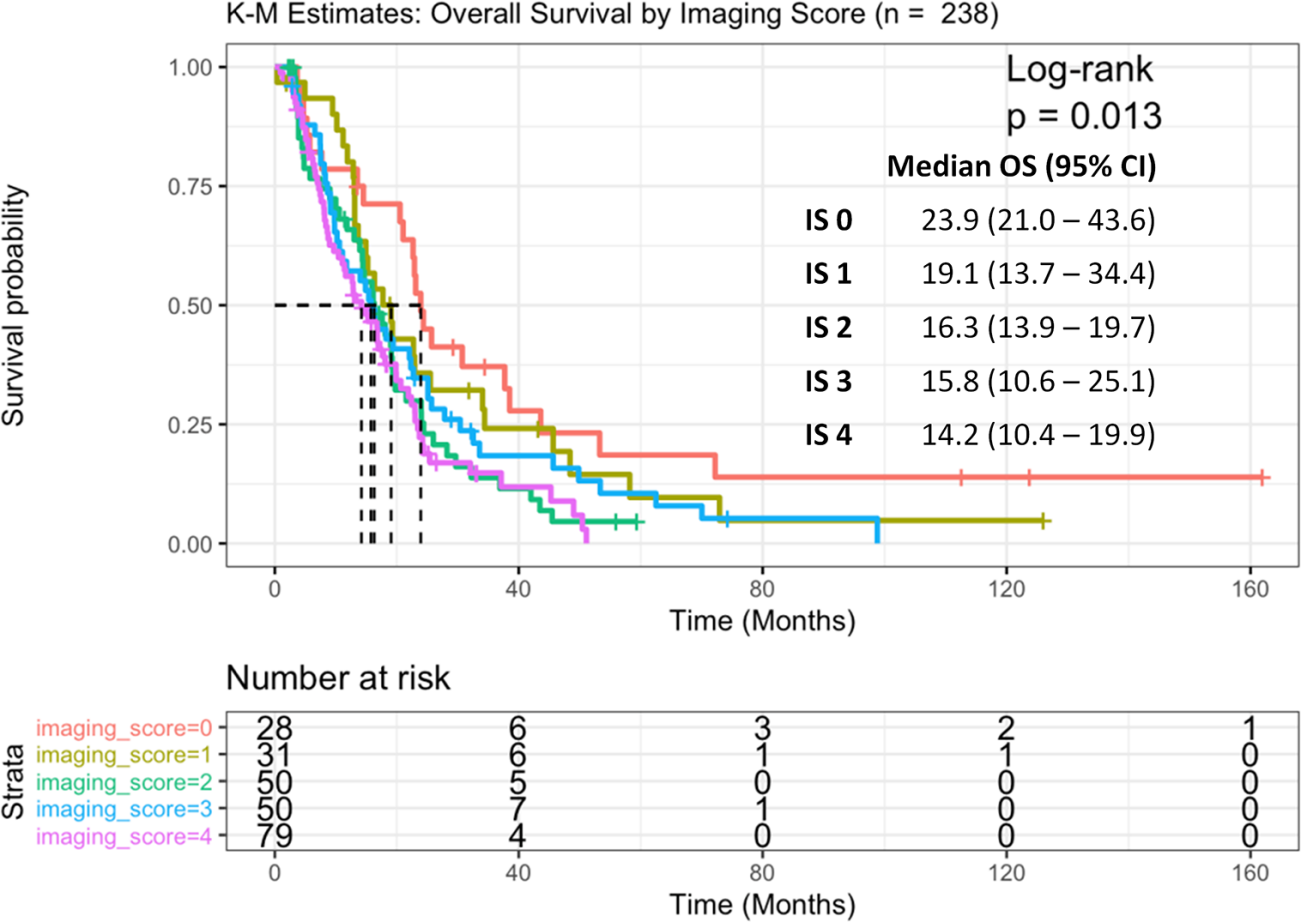
Kaplan Meier curve of overall survival (OS) across all imaging score (IS) subgroups (0,1,2,3,4).

The subgroup of patients with zero PSMA uptake (n = 28) had the best prognosis, with a median OS of 23.9 months (95%CI: 21.0–43.6, p = 0.013), compared to each subgroup with IS 1, 2, 3, and 4, respectively (**Figure 3**). After adjusting for subsequent life-prolonging therapies, which were univariately associated with OS (**Table 4**), and CALGB (Halabi) prognostic factors (17), higher IS was significantly associated with worse OS (HR 1.7, 95% CI 1.2–2.4; p = 0.003) on multivariable Cox regression analysis (**Table 5**).

**Table 4:** Univariate analysis of overall survival according to life-prolonging therapies.

Predictor	Unadjusted Hazard Ratio	Standard Error	95% CI: Lower	95% CI: Higher	P-Value
**Taxanes** **(yes vs. no)**	0.594	0.148	0.444	0.794	<0.001
**ARPI** **(yes vs. no)**	0.392	0.181	0.275	0.559	<0.001
**Radium-223** **(yes vs. no)**	0.243	0.390	0.113	0.523	<0.001

**Table 5:** Multivariate analysis of overall survival.

Predictor	Adjusted Hazard Ratio	Standard Error	95% CI: Lower	95% CI: Higher	P-Value
**Imaging Score (IS)** **high (2-4) vs. low (0-1)**	1.710	0.179	1.204	2.428	0.003
**CALGB (Halabi) Category** **(high vs. low/intermediate)**	1.665	0.156	1.228	2.259	0.001
**Life-Prolonging Therapy** **(yes vs. no)**	0.486	0.152	0.361	0.655	<0.001

**Figure 4:**
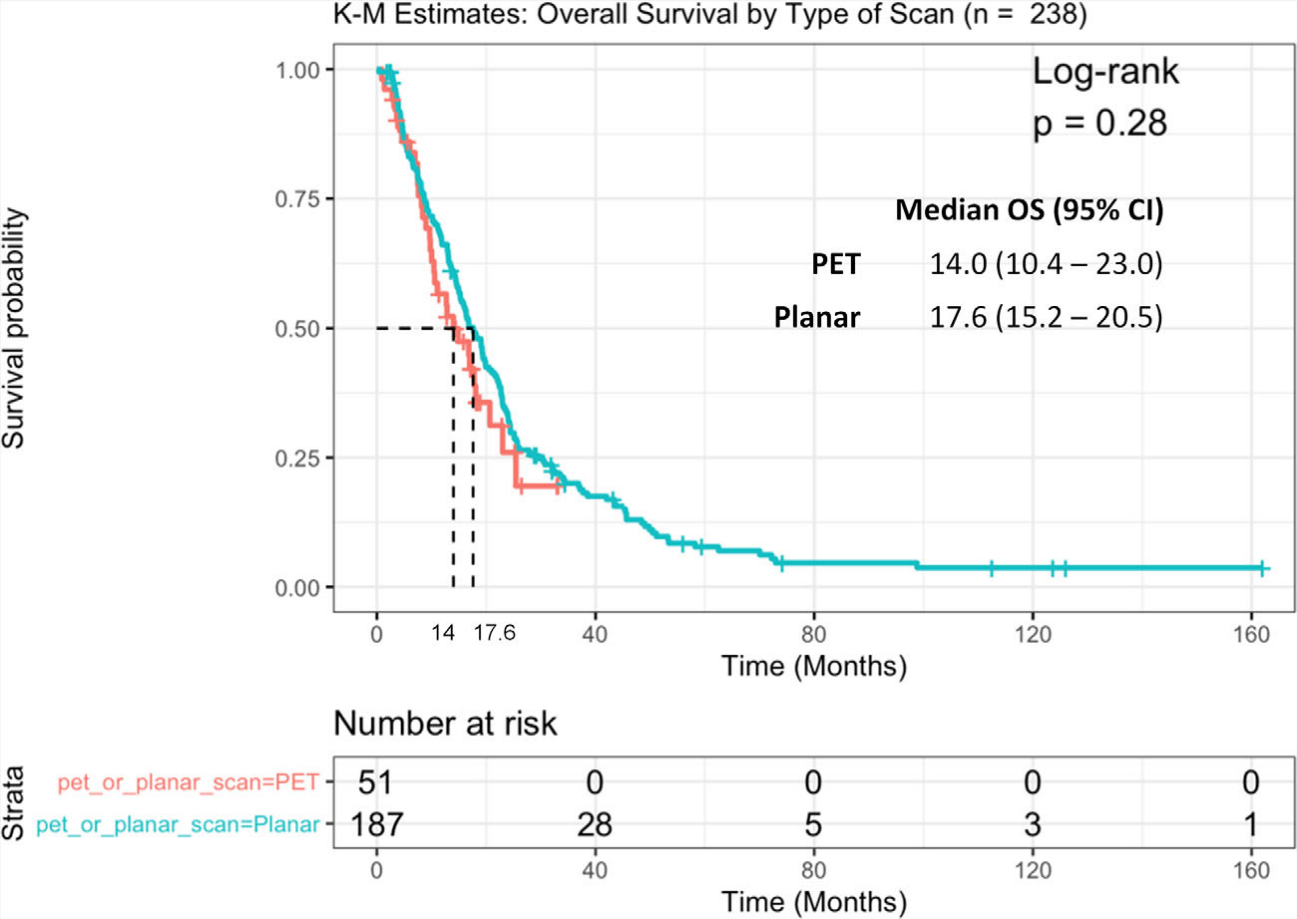
Kaplan Meier curve of overall survival (OS) between patients with PSMA SPECT vs. PET/CT scan.

Because our patient population was assessed with two different PSMA imaging modalities, we examined whether the type of imaging scan (SPECT or PET) had any effect on OS. No statistically significant differences were observed in OS between these two groups, although patients who underwent PSMA-PET tended to have a shorter median OS of 14 months (95%CI: 10.4–23.0) compared to those who had planar imaging [17.6 (15.2–20.5) months, log-rank p = 0.28] (**Figure 4**).

## Discussion

In the present study, we examined the association of noninvasive, radiographic assessment of PSMA expression with OS in patients with mCRPC. We found a significant correlation of high PSMA uptake, represented by IS 2–4, with shorter median OS. This was observed independently of the prognostic impact of life-prolonging therapies that the patients received after PSMA imaging (including taxanes, potent androgen receptor pathway inhibitors, sipuleucel-T, and radium-223) and of the CALGB (Halabi) prognostic risk stratification.

High expression of PSMA in primary and metastatic tumor biopsies and in circulating tumor cells from patients with mCRPC has been associated with worse OS compared to low or absent PSMA expression, in recent studies (7, 20, 21). Besides this association between PSMA and a more aggressive biological behavior, the presence and amount of PSMA avid lesions may reflect the overall burden of disease (i.e., a larger volume lesion with the same cellular expression of PSMA may result in higher imaging uptake). In a meta-analysis of 4790 patients with biochemically recurrent PCa, PSMA-avid lesions on ^68^Ga-PSMA PET correlated with PSA levels and were able to suggest a change in treatment decisions (2, 22). The degree of tracer uptake (SUVaverage and SUVmax) in such patients significantly correlated with PSA plasma levels (23). In hormone-naive metastatic PCa, the effect of a high metastatic burden or bulky disease even on conventional imaging, regardless of slight differences in the definitions of low and high volume across different trials, is a substantial and undisputable predictor of shorter OS (24). Likewise, in patients with mCRPC, a higher number of skeletal metastases (≥5) and the presence of visceral metastases (successive increased lethality for lung and liver, respectively) are both independently associated with worse prognosis (25, 26). ^68^Ga-PSMA PET has been demonstrated to upstage advanced PCa, potentially impacting therapeutic decisions in nonmetastatic CRPC patients (27) as well as in mCRPC patients who were candidates for radium-223, by revealing previously undetected nodal and/or visceral metastases (28).

Our results confirm these observations in principle and further expand them in patients with mCRPC, supporting the notion that real-time, noninvasive semi-quantitative measurement of PSMA by SPECT or PET may serve as a prognostic tool that more accurately represents the overall extent of metastatic burden. A smaller, retrospective study of patients with mCRPC undergoing various life-prolonging systemic therapies (radium-223, cabazitaxel, docetaxel, abiraterone, enzalutamide) assessed the SUVmean, SUVmax, and SUVpeak from PSMA-11 PET/CT and PSMA-11 PET/MRI performed within 8 weeks before and 6 weeks after systemic therapy (29). None of the aforementioned image evaluation parameters were associated with OS, attributed by the authors to the short follow-up time and small number of death events (29). A larger, retrospective study demonstrated increased mortality risk for SUVmax/SUV ratios of lesion-to-liver or lesion-to-spleen, which were higher than their defined cutoffs, respectively (30).

Other cohort studies performed 68Ga-PSMA-11 PET/CT mostly as screening to verify sufficient PSMA expression prior to treatment with PSMA-radioligand therapies (31). In a prospective phase II trial of ^177^Lu-PSMA-617, a combination of fluorodeoxyglucose (FDG)- and PSMA-PET/CT was used to select patients who in theory had a higher likelihood of response to therapy than an unselected population with mCRPC (32). In their univariate analyses for prognostic biomarkers, the mean intensity of PSMA-avid tumor uptake was favorably associated with OS (33). A minority of heavily pretreated patients with low PSMA expression or discordant FDG-avid disease, who were screened for but excluded from treatment with ^177^Lu-PSMA-617, experienced rapid progression and short OS, though we do not know their outcome should they have been treated (34). In a retrospective study of ^177^Lu-PSMA-617-treated patients with available pre-therapeutic ^68^Ga-PSMA-PET/CT scans, the average SUV_max_ of all metastases (PSMA_average_) but not the SUV_max_ of the highest PSMA expressing metastasis (PSMA_max_) was prognostic of OS (35). Adding more complexity, within the subset of patients with high PSMA_average_, those with low minimal PSMA expression (PSMA_min_) had a median OS (11 months) that was intermediate between low PSMA_average_ (5 months) and high PSMA_average_/high PSMA_min_ (21 months) (35). It is important to note that in all these analyses, patients were pre-selected for response to PSMA-TRT (with a high PSMA (target) expression) or for those unexpected to respond (low PSMA expression); thus, distribution of OS may have been skewed. Additionally, the use of FDG-PET/CT in the phase II study of Hofman et al. (32) and its absence from other studies (35), including ours, makes direct comparison tenuous.

Newer PSMA PET metrics, such as whole body tumor volume (36), or the dynamics of a metric change rather than a static value may also be clinically relevant, and potentially superior to those most frequently used, such as SUVmax at baseline. In a small retrospective cohort of 19 patients with mCRPC treated with ^177^Lu-PSMA I&T, while OS rates did not differ between responders and non-responders according to SUVmax, a decrease in either the PSMA tumor volume (PSMA-TV) or the PSMA tumor lesion expression (PSMA-TL) calculated with a semi-automatic program on Ga-68 PSMA PET/CT images pre-and post-treatment was predictive of OS compared to lack of decrease (37). Methodological differences in the measurement of PSMA-avid lesions may account, at least partially, for variability in the reported results. A new molecular imaging TNM (miTNM) staging system, named PROMISE (Prostate Cancer Molecular Imaging Standardized Evaluation), may help harmonize the tools to develop robust prognostic and predictive biomarkers for these patients in the near future (38). Overall, it is plausible that while PSMA expression may be a poor prognostic factor overall, its abundance in metastatic lesions may predict improved outcomes if targeted therapeutically.

This study was limited by the retrospective analysis of prospectively enrolled patients, the uncertainty for cut-offs that may potentially define different levels of PSMA expression, and the use of two different imaging modalities with different detection sensitivities (39). SPECT imaging has poorer resolution compared to PET, and therefore low PSMA expression might not be a straightforward surrogate for smaller volume of disease (and vice versa). However, the majority of patients underwent the same type of imaging (SPECT), and multivariable analysis including known prognostic factors accounting for disease status was consistent, thus reducing the impact of this bias. Additionally, our study population was heterogeneous with respect to subsequent life-prolonging therapies received.

## Conclusion

PSMA represents an important diagnostic and therapeutic target in mCRPC. While its presence has been associated with a higher likelihood of clinical benefit from PSMA-targeted radionuclides, this study assessed the relationship of PSMA imaging expression with overall prognosis of patients with mCRPC, some of whom were subsequently treated with standard therapies with previously demonstrated overall survival benefit. Our findings suggest that a high PSMA imaging score on SPECT or PET/CT is an independent prognostic indicator of poor OS in mCRPC, which is concordant with PSMA’s biological role as a hallmark of lethal, aggressive PCa.

## Data Availability Statement

The raw data supporting the conclusions of this article will be made available by the authors, without undue reservation.

## Ethics Statement

The studies involving human participants were reviewed and approved by Institutional Review Board and Ethics Committee of Weill Cornell Medicine, Cornell University, Weill Cornell Medical Center. The patients/participants provided their written informed consent to participate in this study.

## Author Contributions

Study concept and design: ST and NB. Acquisition of data: PV,MN, MS, JO, AM, DN, NB, and ST. Analysis and interpretation of data: PV, MN, PC, CT, NB, and ST. Drafting of the manuscript: PV, MN, and ST. Critical revision of the manuscript for important intellectual content: PV, JO, AM, DN, NB, and ST. Statistical analysis: CT and PC. Obtaining funding: ST and NB. Administrative, technical, or material support: NB and ST. Supervision: NB and ST. All authors contributed to the article and approved the submitted version.

## Funding

This work was supported by US Department of Defense grants W81XWH-13-PCRP-CCA, W81XWH-09-1-0596, W81XWH-04-1-0267, W81XWH-14-2-0159, and W81XWH-17-PCRP-IA; the Prostate Cancer Foundation; National Institutes of Health grants ULI RR024996, 1-KL2-RR024997-01, and PTBF5405; the David H. Koch Foundation; the Robert Dow Foundation; and the Lawrence and Carol Zicklin Charitable Trust.

## Conflict of Interest

ST certifies that all conflicts of interest, including specific financial interests and relationships and affiliations relevant to the subject matter or materials discussed in the manuscript (e.g., employment/affiliation, grants or funding, consultancies, honoraria, stock ownership or options, expert testimony, royalties, or patents filed, received, or pending), are the following: NB is an inventor of patents assigned to the Cornell Center for Technology Licensing for the J591 antibody described in this article. He is also a paid consultant for and holds equity in BZL Biologics, LLC, the company to which these patents were licensed for further research and development and is a SAB member and holds equity in Telix Pharmaceuticals, Ltd, sub-licensed to develop J591-Lu^177^. ST has served as a paid consultant to Endocyte/AAA/Novartis and Blue Earth, as an unpaid consultant to Atlab and Telix, and Weill Cornell Medicine has received research funding from Endocyte/AAA/Novartis, Progenics, and Atlab/Telix.

The remaining authors declare that the research was conducted in the absence of any commercial or financial relationships that could be construed as a potential conflict of interest.
